# The Effect of Union Status at First Childbirth on Union Stability: Evidence from Eastern and Western Germany

**DOI:** 10.1007/s10680-013-9304-7

**Published:** 2014-01-31

**Authors:** Christine Schnor

**Affiliations:** Interface Demography, Vrije Universiteit Brussel, Pleinlaan 2, 1050 Brussels, Belgium

**Keywords:** Union stability, Separation, Cohabitation, Marriage, Non-marital parenthood, Selectivity, German Family Panel

## Abstract

It is often assumed that cohabitation is much less stable than marriage. If cohabitation becomes more common among parents, children may be increasingly exposed to separation. However, little is known about how the proportion of cohabiting parents relates to their separation behavior. Higher shares of childbearing within cohabitation might reduce the proportion of negatively selected couples among cohabiting parents, which could in turn improve their union stability. This study focuses on parents who were cohabiting when they had their first child. It compares their union stability within a context in which they represent the majority or the minority. The German case is well-suited to this research goal because non-marital childbearing is common in eastern Germany (60 %) but not in western Germany (27 %). The data came from the German Family Panel (pairfam), and include 1,844 married and cohabiting mothers born in 1971–1973 and 1981–1983. The empirical results suggest that the union stability of cohabiting mothers is positively related to their prevalence: survival curves showed that eastern German cohabiting mothers had a greater degree of union stability than their western German counterparts. This difference increased in the event-history model, which accounted for the particular composition of eastern German society, including the relatively low level of religious affiliation among the population. Controlling for unobserved heterogeneity did not change this result. In sum, these findings indicate that context plays an important role in the union stability of cohabiting parents.

## Introduction

In recent decades, non-marital births have increased dramatically (Heuveline and Timberlake 2004; Kiernan 2002, 2004; Perelli-Harris et al. 2012; Sobotka and Toulemon 2008). Most of these births take place within cohabiting unions (Perelli-Harris et al. 2012; Sobotka and Toulemon 2008). In response to this increase, family demographers are devoting more attention to cohabitation as a family context. With regard to relationship stability, many studies have found consistent evidence that cohabiting parents are at higher risk of union dissolution than married parents (Britain: Steele et al. 2006; Germany: Bastin et al. 2012; Norway: Jensen and Clausen 2003; Sweden: Kennedy and Thomson 2010; Canada: Le Bourdais et al. 2000a, b; Le Bourdais and Lapierre-Adamcyk 2004; United States: Manning et al. 2004; Manning 2004; Raley and Wildsmith 2004; Wu and Musick 2008; cross-national studies: Kiernan 2002; Andersson 2002, 2003; Andersson and Philipov 2002; Heuveline et al. 2003; Clarke and Jensen 2004; summarized in Lyngstad and Jalovaara 2010). This higher dissolution rate is commonly attributed to the lack of commitment within cohabiting unions and the negative selection into non-marital family formation. Despite the recent increase in the number of non-marital births, a significant degree of variation in the share of births within cohabitation across western countries remains. To date, however, relatively little is known about how the prevalence of cohabiting births relates to the separation behavior of cohabiting parents.

The issue of union stability is particularly relevant for assessing the implications of the rise in cohabitation for the well-being of children. Changes in the family structure, and especially parental separation, can have negative effects on a child’s future development (Amato 2001; Kim 2011; Kalil et al. 2011), and on a child’s well-being (Osborne and McLanahan 2007). Lone-parent families also tend to have lower incomes than two-parent families (Thomas and Sawhill 2005). To ensure that children have equal opportunities, policymakers have the responsibility to develop measures that can offset the negative effects of separation on children (Mooney et al. 2009). It is often assumed that cohabitation is much less stable than marriage, which would mean that if cohabitation were to become more common among parents, children would be increasingly exposed to the risk of eventually living in single-parent families and step-family arrangements (Osborne and McLanahan 2007; Jensen and Clausen 2003). This may be expected to result in an increase in expenditures on social policies designed to reduce the levels of poverty and the risk of poor well-being among children in separated families. An expansion of cohabitation may also raise the issue of whether governments should support policies that promote marriage. Yet some scholars have taken a very different view of the meaning of this trend, arguing that the rise in childbearing within cohabitation may indicate that cohabitation functions as a stable environment for childrearing that is comparable to marriage (Heuveline and Timberlake 2004; Kiernan 2002; Raley 2001). According to this line of thinking, a further rise in the proportion of cohabiting parents would not be linked to increased demands for governmental support, and policies that promote marriage would be ineffective in reducing child poverty. Thus, the prevalence of non-marital parenthood and the separation behavior of unwed mothers are matters of substantial policy interest.

In a number of recent studies, scholars have suggested that the risk of union dissolution depends on the prevalent union behavior within a specific setting (Le Bourdais et al. 2000a, b; Le Bourdais and Lapierre-Adamcyk 2004; Liefbroer and Dourleijn 2006; Steele et al. 2006; Reinhold 2010). The authors of some of these studies referred to the effect of premarital cohabitation on divorce risks (Liefbroer and Dourleijn 2006; Reinhold 2010). They argued that former cohabitants have a higher risk of divorce in societies in which the majority of couples marry directly, because these couples deviated from the standard path. This idea can be transferred to the context of cohabiting families: if the majority of parents in a given society are married, cohabiting parents should face a higher level of union instability than if cohabitants make up the majority (Heuveline and Timberlake 2004). So far, only a handful of comparative studies have focused on the impact of the share of non-marital childbearing on separation behavior, and have investigated differences across time (Jensen and Clausen 2003) and among cohorts (Steele et al. 2006), countries (Clarke and Jensen 2004), and regions (Le Bourdais et al. 2000a, b; Le Bourdais and Lapierre-Adamcyk 2004). The results of these studies suggest that the importance of commitment via marriage and the strength of negative selection mechanisms into cohabitation could decrease if cohabiting families were to become standard, which could in turn improve the union stability of cohabiting parents. In their empirical analyses, these authors were, however, unable to identify the factors that account for stability differences.

The present study seeks to contribute to this discussion by comparing the union stability of cohabiting parents in a context in which they represent the minority, to the union stability of cohabiting parents in a context in which they make up the majority. Areas or countries in which the majority of all children are born outside of marriage have not yet been studied in a comparative analysis of non-marital union stability. In only few countries, like Estonia and Iceland, are more than half of the children born to non-married parents. However, in the post-socialist eastern part of Germany, the share of non-marital childbearing is 60 %. In western Germany, by contrast, the corresponding share is just 27 %. Thus, the percentage of out of wedlock births in western Germany is not only much lower than it is in eastern Germany; it is also below the European Union average of 37 % (Eurostat, Pötzsch 2012; data from 2010). Since 1990, eastern German mothers have been exposed to the same legal regime as western Germans. However, structural and attitudinal differences between the two parts of Germany have remained, and the gap in non-marital childbearing has actually widened. This persisting regional divide makes Germany an interesting and unique case for comparative analyses. Previous east–west comparisons have been shown to be fruitful for demographic research (Arránz Becker et al. 2010; Konietzka and Kreyenfeld 2002; Rosenfeld et al. 2004).

The present study uses data from the German family panel (pairfam), which includes an eastern German oversample. An advantage of this dataset is that it provides information on the partnership history prior to household formation. The analysis investigates the union stability of couples during the 10 years after the birth of their first child. Event-history methods are applied to the retrospective histories on the partnership dynamics of mothers who were born in the 1970s and 1980s, and who started their families in post-unification Germany. The analyses cover 1,072 western German mothers (288 cohabiting at the first childbirth) and 627 eastern German mothers (384 cohabiting at the first childbirth). Union stability is modeled in a piecewise continuous hazard model in which the union context is considered as an exogenous variable. To analyze the impact of background factors on the risk of separation, a stepwise model procedure is applied. The union context at the first childbirth is brought into the picture with a detailed sample description and a multivariate probit model. The hazard model is then estimated jointly with the probit model (the probability of a first birth within cohabitation), which makes it possible to control for factors that affect the selection into the union status at childbirth.

## Non-marital Family Formation in Eastern and Western Germany

According to the most recent statistics, more than 60 % of all children in eastern Germany are born out of wedlock, compared to 27 % of western German children (Kreyenfeld et al. 2011a; Pötzsch 2012). The differences between eastern and western Germany overshadow other geographical variations (Klüsener and Kreyenfeld 2009). In international comparisons, eastern and western Germany represent nearly opposite ends of the spectrum.

The high eastern German level of non-marital births is surprising in view of the fact that German law provides strong incentives for marital childbearing, like financial benefits (tax advantages, spouse insurance, and alimony rights after divorce) and legal advantages in the case of joint custody and in the recognition of paternity (Konietzka and Kreyenfeld 2002).

Eastern Germany has traditionally had higher shares of non-marital childbearing as well as higher levels of female labor participation, both of which are more accepted in the Protestant than in the Catholic church (Arránz Becker et al. 2010; Klüsener and Goldstein 2012). Moreover, although the region had been dominated by the Protestant church, eastern Germany was strongly secularized in the socialist period, which further weakened the norm of marital childbearing. In addition, socialist East Germany had family policies, such as a special maternal leave program, that privileged non-married mothers (Klüsener et al. 2012; Konietzka and Kreyenfeld 2002). By contrast, West German policies offered financial and legal advantages to married couples with children which were especially beneficial if the wife did not continue to work (Konietzka and Kreyenfeld 2002). In 1990, the legal system of East Germany was replaced by the West German system (Konietzka and Kreyenfeld 2002). However, the share of non-marital childbearing in eastern Germany did not converge to the western German share, but rather increased steadily. Similarly, the levels of secularization and female employment remained high, even though the political pressure of the socialist regime—which strongly discouraged church membership and strongly encouraged the full-time employment of women—no longer existed, and the employment conditions in the region worsened (Kreyenfeld and Geisler 2006). At the individual level, being non-religious and work-oriented have been shown to favor non-marital childbearing in eastern Germany (Kreyenfeld et al. 2011b; Arránz Becker et al. 2010).

Recent statistics have shown that in both western and eastern Germany, women who are not married are most often cohabiting when they have children: among western German women of the birth cohorts 1971–1973, 66 % were married and 20 % were cohabiting at the time their first child was born, while 6 % had a non-coresiding partner and 8 % had no partner. Among their eastern German counterparts, 37 % were married, 43 % were cohabiting, 8 % had a non-coresiding partner, and 12 % had no partner (Bastin et al. 2012).

## Theoretical Considerations, Previous Empirical Findings, and Hypotheses

### Cohabitation and Union Stability

Cohabitation is defined as a non-marital coresiding partnership (e.g., Heuveline et al. 2003). The legal equivalent of cohabitation is marriage. In modern societies, the couples themselves decide whether or not they get married (Cherlin 2004). Thus, the role of cohabitation in a population and its implications for stability have to be discussed relative to marriage. The alternative of having a partnership with separate households will not be considered in the following.

According to exchange theory, relationship stability is determined by the intensity of successful interactions. The more interwoven the interactions are, the more likely the partners are to continue to interact because of the highly specific rewards they can expect from this specific relationship (Thibaut and Kelley 1959: 100ff). This can be described as commitment. Compared to married couples, cohabitants are assumed to be less committed to the partnership, because they have not entered into a formal arrangement (Le Bourdais et al. 2000a). Meanwhile, the legal rights and duties of the cohabiting couples are not (or are to a lesser extent) regulated. Cohabitation and marriage differ, for example, in terms of separation procedures, and in how the disadvantages of separation are balanced (Blossfeld et al. 1999; Perelli-Harris and Sánchez Gassen 2012; Steele et al. 2006). Cohabiting couples also tend to have lower fertility levels than married couples (Oláh and Bernhardt 2008), and a less specialized division of household labor (Brines and Joyner 1999), both of which may reduce the gains from interaction. Cohabitants may also feel less emotionally committed and less socially accepted than married couples (Perelli-Harris et al. 2012). Research has further suggested that cohabiting couples have attitudes that impede strong commitment and enhance separation risks: they are assumed to be more open to the idea of separation, and to be less family oriented, less traditional, and more individualistic (e.g., Le Bourdais et al. 2000b; Lillard et al. 1995, see also Steele et al. 2006; Wu and Musick 2008). Cohabitation can serve as a screening device for marriage, weeding-out matches in which the partners are less compatible (Oppenheimer 1988). The longer the partnership endures, the more the couple learn about their degree of compatibility, which may eventually lead to either marriage or separation (Becker et al. 1977; Brien et al. 2006; Reinhold 2010). Consequently, cohabiting partners were found to be less compatible than married partners (Brien et al. 2006).

Several scholars have pointed out that these characteristics apply to a particular form of cohabitation: namely, that of living together as a testing stage for childless dual-earner couples prior to family and marriage formation. Jalovaara (2013), p. 172 stated that “cohabitors and married persons should be viewed as the same people at successive phases of their family formation processes rather than as representatives of distinct groups.” Thus, previous analyses of the stability differences between marriages and cohabitations often resembled a comparison of apples and oranges. Not surprisingly, a number of studies have found that cohabitations are shorter lived than marriages (Berrington 2001; Heuveline et al. 2003; Hoem and Hoem 1992; Kennedy and Bumpass 2008). Restricting the investigation to first-time parents improves the comparability of the stability levels of cohabitation and marriage, and it ensures that groups, rather than life course stages, are analyzed. In light of the growing share of children born within cohabitation, it seems reasonable to compare fertile cohabitations and marriages. In many respects, families formed by cohabiting parents resemble married families: they are at the same life course stage; and they are headed by two biological parents who presumably share income, housework, and childcare (Wu and Musick 2008).

### Prevalence of Cohabiting Parents and Union Stability

The transition to parenthood increases the level of commitment within the partnership because children represent a union-specific investment (Becker et al. 1977). During this family formation period, many couples marry, while others remain in cohabitation. Because an unborn child can motivate the parents to marry, it is important to focus on the time of the birth of the first child to examine the impact of non-marital parenthood on the risk of separation. In some societies, childbearing within cohabitation is rare, because cohabitation is usually a childless prelude to marriage that ends when the partners are ready to start a family. Being married at the time the first child is born may be important to the parents for normative reasons: i.e., forming a family out of wedlock may violate religious traditions or social norms. Moreover, the tax structure may favor married families, and the establishment of paternity and joint custody may depend on the legal status of the union at childbirth, as has been the case in Germany (Perelli-Harris and Sánchez Gassen 2012; Blossfeld et al. 1999; Ermisch 2005). In other societies, cohabitation is a common alternative to the marital family, and may be chosen by parents as a long-term union form (Heuveline and Timberlake 2004; Perelli-Harris et al. 2012). Parents may choose to remain in cohabitation because they do not expect a significant gain to the partnership from marital childbearing (Seltzer 2000).

Supporters of the idea that the union stability of cohabiting parents is likely to be positively related to the prevalence of such unions point out that the composition of fertile couples living in cohabitation changes as the prevalence of this union form increases. Detractors argue that no such relationship exists, as they assume that the characteristics of cohabiting parents remain similar. In the following, I discuss both lines of argumentation, and derive from them my hypotheses.

On the one hand, a higher share of childbearing within cohabitation can reduce the share of negatively selected couples among cohabiting parents, which in turn improves union stability (Heuveline and Timberlake 2004; Steele et al. 2006). If a couple remain non-married in a society in which cohabitation is not regarded as an appropriate setting for bearing and rearing children, this choice may be related to deficiencies in the partnership (Becker et al. 1977; Blossfeld et al. 1999; Brien et al. 2006; Ermisch 2005; Steele et al. 2006). The couple may be expected to have relatively low gains from their interactions, and their union will be at high risk of dissolution. By contrast, in a society in which non-marital childbearing is common, cohabiting parents will be more heterogeneous with respect to their selectivity. In this case, a couple with a solid partnership and good prospects for stability will continue to cohabit because they see no need to marry (Le Bourdais et al. 2000b; Liefbroer and Dourleijn 2006; Steele et al. 2006; Reinhold 2010). Thus, the union stability of cohabiting parents may be expected to be higher in the second scenario than in the first. There is some empirical evidence that cohabiting parents have worse partnership prospects if the prevalence of their union form is rather low. Several cross-national studies have suggested that the union stability of cohabiting parents is often lower in countries where marital childbearing is common (Andersson 2002; Clarke and Jensen 2004; Heuveline et al. 2003; Le Bourdais et al. 2000a, b; Kiernan 2002; Le Bourdais and Lapierre-Adamcyk 2004). A British study investigated the effect of childbearing within cohabitation across birth cohorts and found that cohabiting mothers born in more recent cohorts were experiencing a higher degree of union stability (Steele et al. 2006).

The high rate of cohabitation among eastern German parents suggests that these cohabitating couples might be less likely to have characteristics that make them prone to separation than their western German counterparts, and that these characteristics could be related to their higher degree of union stability. Recent descriptive research has indeed shown that, while cohabitation is less stable than marriage, eastern German women who cohabited at the time of family formation had better prospects of partnership success than their western German counterparts (Bastin et al. 2012; Perelli-Harris et al. 2012). Thus, the main research hypothesis is that an eastern German mother who was cohabiting when her first child was born will have a lower risk of separation than her western German counterpart.

On the other hand, there is also reason to assume that the union stability of cohabiting parents is fairly independent of the prevalence of childbearing within cohabitation across the society. First, by definition, cohabitation does not involve the formal arrangements associated with marriage. Marriage usually works as a protection of past and future union-specific investments, because it imposes high financial, legal, emotional, and social exit costs. As long as this formal difference between marriage and cohabitation exists even in societies in which non-marital childbearing is common, cohabiting parents may be exposed to the same risk of separation risk as in other settings. Second, cohabiting parents “may be selected on less traditional attitudes about the family, which in turn may be associated with union stability” (Wu and Musick 2008, p. 716). Within a population with high shares of childbearing within cohabitation, non-traditional attitudes—which may, for example, be expressed in high levels of secularization and employment among mothers—might simply be more widespread. Indeed, a Norwegian study (Jensen and Clausen 2003) found that among children born to cohabiting parents, the risk that they would experience a parental break-up remained high over time. Although childbearing within cohabitation was becoming increasingly common, cohabitation was not found to be related to stability for this cohort of children.

The formal differentiation of marriage and cohabitation made by the German state also applies to eastern Germany, which suggests that the separation risks might be similar. The high eastern German shares of non-marital childbearing might simply be a reflection of more liberal attitudes, which are also apparent in the high levels of secularization and maternal employment in eastern Germany. Thus, a competing hypothesis is that an eastern German mother who was cohabiting when her first child was born will have the same separation risk as her western German counterpart.

## Data and Methods

### Selection of the Sample

The analysis was based on data from the German Family Panel (pairfam Release 3.0).^1^ The *Panel of Intimate Relationships and Family Dynamics* provides full fertility and partnership histories of men and women born in 1971–1973, 1981–1983, and 1991–1993 (Huinink et al. 2010; Nauck et al. 2012). These data were supplemented by DemoDiff (Release 2.0), an oversample of eastern German respondents born in the years 1971–1973 and 1981–1983 (Kreyenfeld et al. 2011b). The data were gathered between 2008 and 2011, approximately 20 years after reunification.

The present study used a ready-to-use event-history dataset that incorporates the retrospective partnership and fertility histories, which were compiled in the first wave and were updated in the two subsequent waves (Schnor and Bastin 2013). While the partnership information gathered in the survey is very detailed, no information on the individual characteristics of former partners was collected.

The analysis was restricted to women of the birth cohorts 1971–1973 and 1981–1983. The cohorts 1981–1983 were still young at the interview dates, but the event-history approach used in this study took into account the different lengths of time at risk due to age differentials at the time of the interviews. However, mothers of the 1981–1983 birth cohorts represented a more selective population than mothers of the 1971–1973 cohorts, as can be seen from Table 1. The east–west differences found among the younger birth cohorts were in line with those found in other studies, which showed that eastern Germans transitioned to parenthood at younger ages than western Germans (e.g., Arránz Becker et al. 2010; Kreyenfeld et al. 2011a).

**Table 1:** Proportion of mothers relative to all women of the same birth cohorts who participated in the respective waves and reported having at least one biological child, in percentages

	Wave 1 (2008/2009) (%)	Wave 2 (2009/2010) (%)	Wave 3 (2010/2011) (without DemoDiff) (%)
Western Germans			
1971–1973 birth cohort	81.2	83.5	83.6
1981–1983 birth cohort	38.2	43.4	45.0
Eastern Germans			
1971–1973 birth cohort	85.5	87.2	87.7
1981–1983 birth cohort	52.0	56.1	55.7

*Sources* pairfam/DemoDiff (2008–2011)

We concentrated on women who were in a residential relationship with the biological father of their child when they became a mother. Single mothers were excluded from the analysis. None of the women studied was previously married. Married women whose spouse was not the biological father of the child were not considered. To enhance the explanatory power of the regional information, the analysis focused on respondents whose place of birth *and* place of residence at the time of the interview were in the same German region (eastern vs. western Germany).^2^ Internal migrants and foreign-born women were not considered in the analysis, because this would entail obtaining information about the date of migration, and having to make distinctions between the roles played by socialization and by the current environment. Only women who had their first child after reunification (that is, after October 1990) were considered, because the women who became mothers before that date were exposed to different legal regimes. Individuals with inconsistencies in their fertility or partnership histories were omitted from the analysis. The final size of the analytic sample was 1,200 western German and 644 eastern German women (Table 2).

**Table 2:** Description of sample selection

	Sample size (respondents)
Initial sample	13,891
After exclusion of	
Men	7,129
Birth cohort 1991–1993	4,990
Migrants	3,872
Childless persons	2,449
Mothers without coresiding partner at 1st childbirth	2,018
Inconsistencies/unions ending with partner′s death/homosexual unions	1,877
Women who had a 1st birth prior to 10/1990	1,844

*Sources* pairfam/DemoDiff (2008–2011), own estimates

### Method and Analytical Procedure

In this section, I present an empirical model of the risk of separation that incorporated the direct effect of having formed a family within cohabitation and the potential selectivity of separation-prone cohabiting parents. Hazard regressions were used to estimate the relative risks of separation after a couple had their first child. The first separation after family formation was considered to be the event. The observation was censored 10 years after the birth of the couple’s first child, and with the time of the latest interview. The hazard function *h*(*t|X*) consisted of the baseline hazard (*β*_0_(*t*))—defined as a piecewise linear spline with knots 2 and 6 years after the child was born—as well as sets of time-constant and time-variant covariates (*X* and *X*(*t*)) and its vectors of the corresponding parameters (*β*_1_ and *β*_2_). The direct effect of having cohabited with the partner at the time the first child was born on the hazard of dissolution was measured by *β*_3_, the effect associated with COH, as illustrated in the following equation (without observation subscript *i*):

1 $$ {\text{Hazard model }}\left( {\text{transition to separation after first childbirth}} \right) :\;{\text{In}}\,\;h(t|X) = \beta_{0} (t) + \beta_{1} X + \beta_{2} X(t) + \beta_{3} {\text{COH}} . $$

In order to investigate the role of selection, I applied in a first step a stepwise modeling strategy, which allowed me to observe compositional effects in the central covariates of interest. In a second step, I estimated a multi-process model, as suggested by Lillard et al. (1995) and explained in Lillard and Panis (2003), which allowed for the transition to separation after giving birth to be correlated with the selection into the union context when the couple had their first child. A probit function determined the probability of cohabitation at the time of the first childbirth.

The selection into childbearing within cohabitation was considered in a probit model, because this made it possible to compare the characteristics of married and cohabiting mothers in the sample. Among the advantages of using a multi-process model was that the determinants influencing the probability of giving birth while cohabitating could be compared with the determinants of separation, and that causal effects could be disentangled from selection effects.

The unit of observation was the partnership in which the family was formed. This approach enabled me to estimate the influence of unobserved partner-specific characteristics in the absence of available partner information. To identify the correlation structure, it was important to formulate exclusion restrictions; i.e., variables that entered one process but not the other, as there was only one spell per event (see Sect. 4.3). In detail, the following equations (illustrated here without observation subscript *i*) were estimated:

2 $$ {\text{Hazard model }}\left( {\text{transition to separation after first childbirth}} \right):In\,\,h(t|X) = \beta_{0} (t) + \beta_{1} X + \beta_{2} X(t) + \beta_{3} {\text{COH}} + \varepsilon $$

3 $$ {\text{Probit model (probability of a first birth within cohabitation }}\left( {{\text{vs}} . {\text{ marriage}}} \right) :\;{\text{COH}}^{ * } = \alpha_{o} + \alpha_{1} Z + \delta $$

4 $$ {\text{COH}} = \left\{ {\begin{array}{*{20}c} {1\,\,\,{\text{if}}\,{\text{COH}}^{*} \, > \,0} \\ {1\,\,\,{\text{if}}\,{\text{COH}}^{*} \, \le \,0} \\ \end{array} } \right.. $$

The hazard model was complemented by a residual term (*ε*). For the probit equation, *α*_0_ represents the intercept, *Z* represents the independent variables that influenced the probability of having a first child within cohabitation rather than within marriage, with *α*_1_ being the parameters, and *δ* being an unobserved factor. It was assumed that the residuals had a mean value of zero and followed a bivariate normal distribution, where $\sigma_{\varepsilon }^{2}$ and $\sigma_{\delta }^{2}$ denoted the variances of the residuals and $\sigma_{\varepsilon \delta }$ was the covariance between the residuals. In line with other studies that relied on single-spell data (Impicciatore and Billari 2012; Baizán et al. 2003, 2004), the variances of the residuals were fixed to the unity (see also Rabe-Hesketh and Skrondal 2008: 110). The variance of *ε* was then allowed to vary in order to test the robustness of the results. A significant correlation between the residuals means that common unobserved factors influenced both decisions.

5 $$ {\text{Heterogeneity components}}:\;\left( {\begin{array}{*{20}c} s \\ \delta \\ \end{array} } \right)\sim \left( {\left( {\begin{array}{*{20}c} 0 \\ 0 \\ \end{array} } \right),\left( {\begin{array}{*{20}c} {\sigma_{\varepsilon }^{2} } & {\sigma_{\varepsilon \delta } } \\ {\sigma_{\varepsilon \delta } } & {\sigma_{\delta }^{2} } \\ \end{array} } \right)} \right). $$

I used STATA 11.0 for data preparation and descriptive statistics (Blossfeld et al. 2007); the multivariate analyses were performed with the help of the statistical package aML 2.9 (Lillard and Panis 2003).

### Background Variables

The exogenous variables are presented in Table 3. The composition of the sample is shown separately for eastern and western Germans. Table 3 is further subdivided according to the marital status of the couple when they had their first child. Information on the significance levels of regional differences has been added. The number of cases shows that childbearing within cohabitation was more prevalent in the east than in the west. About 60 % (*N* = 385) of eastern German mothers were cohabiting when they gave birth to their first child, compared to 27 % (*N* = 324) of western Germans.^3^,^4^

**Table 3:** Sample composition by region and union form at the time the first child was born

Union form at first childbirth	Sample composition									
	Cohabiting				Married					
	Western Germans		Eastern Germans	*t* test^a^	Western Germans	Eastern Germans			*t* test^a^	
Distribution of respondents, in column percent										
Educational level				**					***	
Low educated	26 %		7 %		19 %	3 %				
Middle educated	35 %		62 %		42 %	61 %				
High educated	38 %		29 %		39 %	36 %				
Missing Information	<1 %		<1 %		<1 %	–/–				
Religious affiliation				***					***	
Catholic	35 %		3 %		43 %	4 %				
Protestant	44 %		17 %		39 %	25 %				
No church member	18 %		79 %		11 %	69 %				
Other affiliation	3 %		<1 %		7 %	2 %				
Missing information	–/–		<1 %		<1 %	–/–				
Living together with both parents until age 18^b^				n.s.					n.s.	
Yes	55 %		57 %		64 %	62 %				
No	21 %		23 %		13 %	21 %				
Missing information	24 %		20 %		23 %	18 %				
Family formed under				*					***	
Old legislation (prior 7/1998)	21 %		25 %		26 %	36 %				
New legislation (after 7/1998)	79 %		75 %		74 %	64 %				
Employment status 9 months prior to first childbirth^c^				***					***	
Non-employed	10 %		15 %		7 %	11 %				
Full-time employed	40 %		34 %		43 %	47 %				
Part-time employed	5 %		10 %		4 %	9 %				
Missing information	45 %		41 %		46 %	35 %				
Sex of first child				n.s.					n.s.	
Male	56 %		53 %		49 %	54 %				
Female	44 %		48 %		51 %	46 %				
Health status of first child				n.s.					***	
Not handicapped	85 %		89 %		83 %	92 %				
Handicapped	15 %		11 %		17 %	8 %				
Season of birth of child				n.s.					n.s.	
Non-winter	51 %		48 %		54 %	55 %				
Winter	49 %		52 %		46 %	45 %				
Number of siblings				**					***	
No Siblings	21 %		25 %		14 %	30 %				
1 Sibling	42 %		48 %		42 %	47 %				
2 or more siblings	37 %		27 %		44 %	23 %				
Birth cohorts				***					n.s.	
1971–1973	70 %		53 %		80 %	78 %				
1981–1983	30 %		47 %		20 %	22 %				
Mean values of time-constant partnership covariates (standard deviations in brackets)										
Age at first childbirth (years)										
Cohorts 1971–1973	28.2 (0.33)		26.2 (0.31)	***	28.0 (0.16)	26.4 (0.29)			***	
Cohorts 1981–1983	22.6 (0.29)		23.2 (0.20)	n.s.	24.3 (0.19)	24.8 (0.29)			n.s.	
Union duration (years)	3.6 (0.19)		4.5 (0.16)	***	6.1 (0.13)	6.0 (0.21)			n.s.	
Partnership order	2.3 (0.07)		1.7 (0.05)	***	2.0 (0.04)	1.6 (0.05)			***	
Relative exposure time in percent of total person months, time-variant partnership covariates and baseline variable										
Employment status^c^				***					***	
Non-employed	23 %		18 %		23 %	12 %				
Full-time employed	10 %		24 %		7 %	28 %				
Part-time employed	18 %		16 %		21 %	21 %				
Missing information	49 %		41 %		49 %	40 %				
Age of first child (baseline)				n.s.					n.s.	
0–1 years	33 %		32 %		29 %	28 %				
2–5 years	45 %		44 %		44 %	43 %				
6 years and more	22 %		24 %		27 %	29 %				
Further biological children				***					***	
No further child	51 %		69 %		43 %	58 %				
One further child	37 %		28 %		48 %	37 %				
Two or more further children	12 %		3 %		10 %	5 %				
Number of subjects	324		385		876	259				
Total exposure time	21,878		27,387		68,870	21,412				
Number of separations	87		92		111	40				

*Sources* pairfam/DemoDiff (2008–2011), own estimates. Weighted by sample design weight (including corrections for birth cohort and place of residence)

Significance levels *** Pr (|*T*| > |*t*|) < 0.01; ** Pr (|*T*| > |*t*|) < 0.05; * Pr (|*T*| > |*t*|) < 0.10

^a^Two-sample *t* tests with unequal variances

^b^Evaluated in wave 2 (pairfam/DemoDiff)

^c^Evaluated in wave 3 (Pairfam), wave 2 (DemoDiff)

**Table 4:** Variable selection in the probit and hazard models

	Cohabiting at childbirth (probit model)	Separation (hazard model)
Educational level	X	X
Religious affiliation	X	X
Living together with both parents until age 18^a^	X	X
Period of family formation	X	X
Employment status 9 months prior to 1st childbirth^b^	X	
Sex of first child		X
Health status of first child		X
Season of birth of child		X
Number of siblings	X	
Birth cohorts	X	X
Age at first childbirth (years)	X	X
Union duration (years)	X	X
Partnership order	X	X
Employment status^b^		X
Age of first child (baseline)		X
Further biological children		X

*Sources* pairfam/DemoDiff (2008–2011), own estimates. Weighted by sample design weight (including corrections for birth cohort and place of residence)

^a^Evaluated in wave 2 (pairfam/DemoDiff)

^b^Evaluated in wave 3 (Pairfam), wave 2 (DemoDiff)

#### *Age of the First Child (Baseline)*

Previous studies found that the risk of separation was reduced, especially in the years immediately following the birth of the first child (Andersson 2002; Hoem and Hoem 1992; Oláh 2001). In the sample, the majority of the exposure time refers to the period when the first child was of preschool age.

#### *Birth Cohort*

The consideration of birth cohorts is important because of sample issues. Women of the younger birth cohorts (1981–1983) were more prevalent in the eastern German sample, but only among cohabiting mothers.

#### *Education*

Research suggests that the educational background is related to the union status at childbirth and to the separation behavior of parents (McLanahan 2004): less educated mothers may be less likely to marry because they see their romantic partners as economically or socially unsuitable marriage partners. They highly value marriage, but believe that their partnership does not meet the high standards they associate with a stable marriage (Edin and Reed 2005). The levels of school education were broken down into three categories: low (no certificate or lower secondary education), middle (secondary education), and high (high school diploma). Respondents with information on school education were assigned to a separate category. Mothers with low levels of school education are rather uncommon in eastern Germany: the vast majority of eastern German mothers have middle or high educational levels (Konietzka and Kreyenfeld 2002). The composition further reveals that mothers who were cohabiting when they had their first child were somewhat less educated than married mothers in western as well as in eastern Germany.

#### *Partnership Duration*

The partnership duration prior to giving birth is the length of the partnership from the time it was formed until the couple had their first child. It may capture the level of positive selectivity, or the weeding-out effect; and it may indicate the maturity of the couple (Manlove et al. 2012). Research has shown that couples who become parents rapidly have lower levels of union stability (Hoem and Hoem 1992; Oláh 2001). Among western German cohabiting couples, the mean partnership duration prior to having a first child was 3.6 years, which was significantly shorter than among eastern Germans, whose mean union duration prior to starting a family was about 4.5 years. Eastern and western German married mothers had a mean partnership duration of 6 years before their first child was born.

#### *Age at First Childbirth*

Being young when the union or family was formed can result in a poor match: young people tend to be less mature and less future-oriented with regard to their partner choice (Becker et al. 1977). They may have also had insufficient time to search for the right partner, and they may have access to attractive alternative candidates on the partner market (Becker et al. 1977; South 1995; Lyngstad and Jalovaara 2010). In the table, the mean ages at which the women first gave birth are shown by birth cohorts. Irrespective of the union context, eastern German mothers born in 1971–1973 were about 2 years younger when they had their first child than western German mothers. Mothers born in 1981–1983 were slightly older in eastern than in western Germany. The age at which a couple had their first child was considered as a metric variable that entered the multivariate estimations linear and squared.

#### *Religious Affiliation*

It has been shown that church members have higher rates of marital childbearing and more stable families (Brüderl et al. 1997; Hoem and Hoem 1992; Lehrer 2004; Oláh 2001). The respondent’s religious affiliation was categorized as Catholic, Protestant, non-affiliated, and other affiliation. The share of non-affiliated women was much higher among eastern Germans, which is attributable to the secularization policy of the GDR. In addition, there were differences by marital status when giving birth: women with no religious affiliation were more likely to have been living in a cohabiting union when they became mothers.

#### *Employment Status*

Female employment has been found to increase the risk of separation, at least among married couples, while the evidence with regard to cohabiting couples has been mixed (Jalovaara 2013; Lyngstad and Jalovaara 2010). The employment status was constructed based on the self-assessed employment history gathered in the third pairfam wave (and in the second DemoDiff wave, respectively). I distinguished between episodes of full-time employment, part-time employment, and non-employment. Episodes of full-time education, unemployment, and home-making were included in the last category. The information was missing if the respondent did not reply or did not participate in the respective waves. Two variables provided information about the employment status: (1) a time-constant variable that showed the employment status 9 months prior to the first childbirth, and (2) a time-variant variable that showed the current activity status after the first child was born. Most of the women for whom information was available had been in full-time employment before their entry into motherhood. After the women gave birth, most of the exposure time was still spent in full-time employment among eastern German mothers. Western German mothers spent most of their time in non-employment.

#### *Partnership Order*

The partnership order was defined as the number of partners, including the partner at the time the child was born. Respondents were asked in the interview to provide information about partnerships that involved co-residence, that lasted longer than 6 months, or that were of personal importance for the respondent. Eastern Germans had a lower mean number of partnerships than western Germans, which held for both married and cohabiting unions. In both regions, cohabiting women reported having more partnerships prior to having their first child than married women. In previous studies, the cohabitation order was shown to have no effect on stability, while higher order marriages were found to be less stable than first marriages (Manlove et al. 2012; Poortman and Lyngstad 2007; Steele et al. 2006). So far, there has been no evidence on the effect of the order of partnerships.

#### *Further Children*

The number of biological children born to a couple has been shown to reduce the risk of separation (Hoem and Hoem 1992; Oláh 2001). Eastern German mothers spent most of the observation time in one-child families. Higher order births were more common among western German mothers.

#### *Living with Both Parents Until Age 18*

People who have experienced parental separation have been shown to be more likely to separate themselves (Lyngstad and Jalovaara 2010). In the second pairfam wave (2009/2010), respondents were asked whether they had lived with both biological parents until they reached age 18. Respondents who did not continuously reside with both parents experienced episodes of living with only one parent or in alternatives arrangements. Respondents who did not participate in this wave or who did not answer the question were grouped into one category. The proportions of women who lived with both parents until their 18th birthday were comparable in western and eastern Germany; western German married women were the least likely to have experienced alternative living arrangements during their childhood and adolescence.

#### *Policy Period*

The date of family formation provides information about how custody for non-married parents was regulated during the respective period. Fathers who were not married did not have the legal right to file for joint custody of their children unless the children were born after July 1998. The legal regulations might have influenced the probability of having had a first birth within cohabitation: children who were born before the policy reform should been more likely to have been born into marriages because of the legal disadvantages associated with non-marital childbearing for fathers. It is also possible that the inability of non-married fathers to secure custody prior to 1998 increased the stability of their partnerships.

#### *First Child’s Characteristics*

Having a daughter has been found to be associated with a higher risk of union disruption (Morgan et al. 1988), as is having a child with disabilities or cognitive delays (Hartley et al. 2010; Hatton et al. 2010; Sobsey 2004). A child was classified as handicapped if he or she had a chronic disease, developmental disabilities, or a physical handicap. Previous studies found a seasonality among wanted births driven by women who have a preference for a non-winter birth (Bobak and Gjonca 2001). The sample composition indicates the proportion of winter births (i.e., the first child was born in September–February). This should serve as an indicator of an unplanned pregnancy, which is related to a higher risk of union disruption (Manning et al. 2004).

#### *Number of Full Siblings*

The size of her family of origin can restrict the amount of resources that are available to a woman, and can therefore negatively influence her decision to marry, because marriage requires more resources than cohabitation. In eastern Germany, cohabiting women indeed had more siblings than married women, but the average size of the family of origin was smaller in eastern than in western Germany, where more women had two or more siblings.

Table 4 shows whether the respective variables were included in both models. Most of the control variables are expected to influence the likelihood of a birth within cohabitation and union stability, and are therefore considered in the probit model as well as in the hazard model. The model specification has exclusion restrictions; i.e., variables that enter one process but not the other. As consecutive events were analyzed, the hazard model includes information that becomes relevant only after childbirth. Beyond time-varying information on the number and the age of the children and the economic activity, this also includes the first child’s characteristics. According to Impicciatore and Billari (2012) and Lillard and Waite (1993), the number of siblings may be assumed to affect only the probability of cohabitation and marriage, but not of dissolution risks. The probit model additionally accounts for the employment status prior to family formation.

In sum, is there any indication that eastern German cohabiting mothers had characteristics that made them less prone to separation than their western German counterparts? When we compare eastern to western German cohabiting mothers, we can see that, on average, the former had more education and a longer union duration prior to childbirth, both of which are factors known to increase union stability. On the other hand, the eastern Germans were younger at the birth of their first child, and they had fewer subsequent children, which are factors associated with lower stability levels. Furthermore, eastern German cohabiting mothers were much less religious than their western German counterparts, and were more likely to have been in full-time employment after entering motherhood. As a consequence, the separation risk did not appear to differ based on the characteristics of mothers who cohabited in eastern and western Germany.

## Empirical Findings

### Descriptive Results

**Fig. 1:**
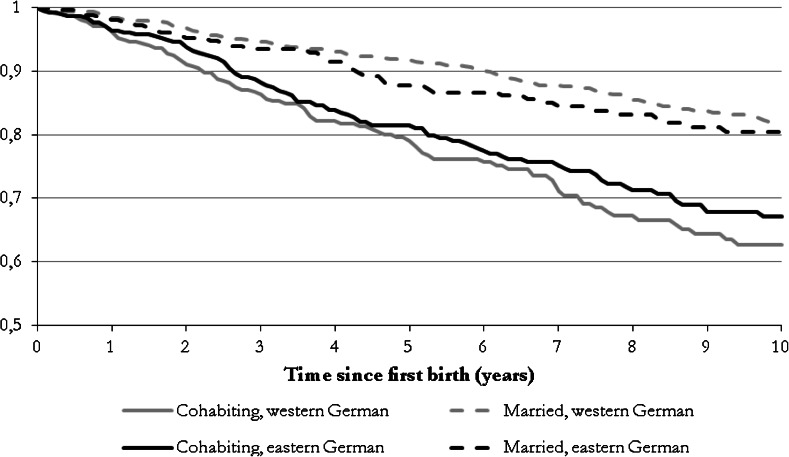
Results of Kaplan–Meier estimates (proportion of women who remained partnered with the child’s father 10 years after having their first child, by union form at the time they had their first child and region. *Sources* pairfam/DemoDiff (2008–2011), own estimates Weighted by sample design weight (including corrections for birth cohort and place of residence). Results of the Cox test (modified log-rank test) for equality of the survival curves of eastern and western German women: no statistically significant differences between cohabiting women (Pr > *χ* ^2^ = 0.30); no statistically significant differences between married women (Pr > *χ* ^2^ = 0.40)

Figure 1 displays the Kaplan–Meier survival estimates (Blossfeld et al. 2007). It provides some initial insights into the transition to separation among eastern and western German mothers, by the age of the first child. It is obvious that in both regions women had higher separation probabilities at all ages of the first child if they were cohabiting rather than married when they gave birth to the child. The Kaplan–Meier survival curves suggest that union stability was somewhat higher for cohabiting couples in eastern Germany than in western Germany. However, the difference was not shown to be significant in the Cox test (specified log-rank test; see StataCorp 2011, p. 447).

#### Stepwise Multivariate Models

The multivariate analysis first followed a stepwise modeling strategy that relied on the hazard model. Model 0 focused on the differences in the separation risks of eastern and western German mothers in general. In Model 1, information on the union form at the time the first child was born was added. Model 2 further considered observed factors that may be related to the match quality of the partnership: the level of school education, the age when the first child was born, and the duration of the union prior to having a child. In Model 3, control covariates were added that account for non-traditional attitudes, including religious affiliation and economic activity. In Model 4, the probit and the hazard model were estimated as separate processes. The hazard model included further variables: the partnership order, the family size (in terms of the number of biological children), the experience of parental separation (expressed in a failure to live with both biological parents until age 18), the first child’s characteristics (sex, health status, season of birth), and a dummy variable indicating whether the child was born before the custody reform in 1998. Finally, the influence of unobserved heterogeneity was estimated in Model 5. Table 5 shows the results of the stepwise models without interaction (Models 0–5a).

**Table 5:** Transition to the first separation after the first child was born, results from a piecewise linear model

	Model 0	Model 1a	Model 2a	Model 3a	Model 4a		Model 5a	
	Hazard e^β^	Hazard e^β^	Hazard e^β^	Hazard e^β^	Probit β	Hazard e^β^	Probit β	Hazard e^β^
Baseline (β)								
Intercept	−6.73***	−6.43***	−4.11**	−4.18**	5.4001***	−3.8878**	7.0179***	−3.6483*
1st child 0–1 years (slope)	0.032***	0.032***	0.030***	0.030***		0.0400***		0.0461***
1st child 2–5 yrs (slope)	−0.002	−0.002	−0.004	−0.004		0.0020		0.0058
1st child 6 years and older (slope)	−0.000	−0.000	−0.004	−0.004*		−0.0025		−0.001
Birth cohorts								
(Ref = 1971–1973)								
1981–1983	2.10***	1.77***	1.15	1.19	0.22**	0.90	0.24**	0.90
Region								
(Ref = Eastern Germany)								
Western Germany	0.85	1.06	1.02	1.50**	−0.75***	1.71***	−1.01***	1.64**
Union form at 1st childbirth								
(Ref = Cohabiting)								
Married		0.49***	0.61***	0.64***		0.67***		0.91
Educational level								
(Ref = Middle)								
Low			1.08	1.12	0.23**	1.06	0.29**	1.14
High			0.79	0.81	0.01	0.87	0.07	0.88
Missing information			2.52*	2.62*	1.26	2.99**	1.26	2.62*
Union duration prior to 1st childbirth			0.90***	0.90***	−0.04***	0.90***	−0.05***	0.88***
Age at 1st childbirth			0.93	0.91	−0.38***	0.92	−0.48***	0.87
Age at 1st childbirth^2^			1.00	1.00	0.01***	1.00	0.01***	1.00
Religious affiliation								
(Ref = Protestant)								
Catholic				0.90	−0.10	0.94	−0.10	0.87
No church member				1.59***	0.19**	1.63***	0.27**	1.73***
Other affiliation				0.63	−0.74***	0.65	−0.96***	0.55*
Partnership order					0.10***	0.97	0.12***	0.99
Living together with both parents until 18th birthday								
(Ref = Yes)								
No					0.14	1.34**	0.21**	1.48***
Missing information					0.11	0.98	0.19*	1.03
Family formed								
(ref = After 7/1998)								
Prior to 7/1998					−0.36***	0.74	−0.53***	0.71
Economic activity after 1st childbirth								
(Ref = Full-time employed)								
Non-employed				0.68***		0.83		0.91
Part-time employed				0.73*		0.79		0.81
Missing information				0.86		1.02		1.09
Economic activity 9 months prior to 1st childbirth								
(Ref = Full-time employed)								
Non-employed					0.18		0.24	
Part-time employed					0.06		0.13	
Missing information					0.05		0.09	
Sex of first child								
(Ref = Male)								
Female						0.96		0.98
Health status of first child								
(Ref = Not handicapped)								
Handicapped						1.03		0.99
Season of birth of child								
(Ref = Winter)								
Non-winter						0.85		0.83
Number of siblings								
(Ref = No Siblings)								
1 sibling					0.02		0.06	
2 or more siblings					−0.04		−0.05	
Covariance $\sigma_{\varepsilon \delta }$ (β)							0.48***	

*Sources* pairfam/DemoDiff (2008–2011), own estimates

Significance levels *** *p* < 0.01; ** *p* < 0.05; * *p* < 0.10

The multivariate results of Models 1a–4a in Table 5 demonstrated that women who were cohabiting when they had their first child had a significantly higher risk of union disruption. None of the observed characteristics could explain this finding. However, when a correlation between the union context at the time of the first childbirth and subsequent union stability was allowed for in Model 5a, the effect of marital status turned out to be insignificant. The increased risk of union disruption for women who cohabited at the time their first child was born could be entirely attributed to the selection of the most separation-prone into cohabitation.^5^

The multivariate results shed more light on the question of whether the high rate of cohabitation in eastern Germany was related to a higher degree of union instability among eastern German mothers. Model 0 showed that western and eastern German women did not differ in their levels of union stability after they had their first child. This result did not change when the union context at the time the first child was born was taken into account in Model 1a, and control covariates for school education, age at first childbirth, and union duration were added in Model 2a. When the higher risks of separation among religious non-affiliated and full-time employed women were taken into account in Model 2-a, western German mothers were shown to have had significantly lower levels of partnership stability than eastern German mothers. Separate estimations indicated that this change was attributable to the significant influence of the mother’s religious background (results not shown). This result was not changed by controlling for the number of children, parental separation, or partnership order in Model 4a, or by accounting for unobserved heterogeneity in Model 5a. In sum, these results revealed that in eastern Germany mothers did not have lower levels of union stability, even though the prevalence of births within cohabitation was much higher than in western Germany. If the religious composition had been the same in the two regions, eastern German mothers would even had a higher degree of union stability than their western German counterparts.

The results from the probit model in Model 4a revealed that women had higher probabilities of giving birth to their first child within cohabitation if they belonged to the younger birth cohorts (1981–1983), were living in eastern Germany, and had a low level of school education; and also if they started their family at a young age or after the policy reform in July 1998. Having no religious affiliation or a short union duration prior to the birth of the first child increased the probability of cohabiting at the time the first child was born, and the risk of separation after family formation. The higher separation risks of cohabiting women could not be explained by these determinants. The mother’s age when she gave birth, her educational level, and the number of her partnerships seemed to have had no significant influence on the stability of her partnership. Having lived apart from a parent during childhood or adolescence and having a one-child family increased the risk of separation among the mothers. Mothers in full-time employment had a lower degree of union stability in Model 3a, but this could be fully explained by controlling for family size in Model 4a.

### Interaction Results

**Fig. 2:**
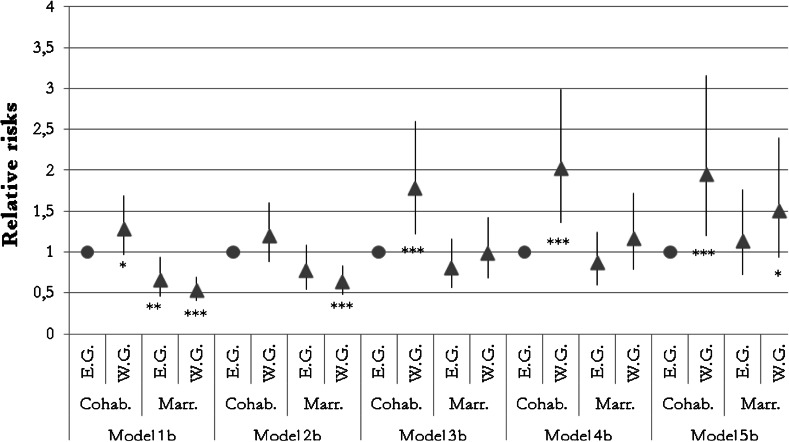
Results of an interaction of region and union form at the time the first child was born within the hazard Models 1b–5b, shown in relative risks with 95 % confidence intervals and significance levels. *Sources* pairfam/DemoDiff (2008–2011), own estimates. Significance levels ***p < 0.01; **p < 0.05; *p < 0.10. *E.G.* Eastern German women, *W.G.* Western German women, *Cohab.* cohabiting at first childbirth, *Marr.* married at first childbirth

The interaction results of the region and the union context at the birth of the first child are shown in Fig. 2. Control covariates were again added stepwise to the models to find the mediating effects (Models 1b–5b). Eastern German women who cohabited when their first child was born formed the reference category.

The interaction terms revealed differences in the separation risks of eastern and western German women who were cohabiting, with the latter having an elevated risk of union disruption. In Model 1b, this difference was only weakly significant (*p* < 0.10). After the lower level of school education, the shorter union duration prior to family formation, and the older age at childbirth of the average western German cohabiting woman were accounted for in Model 2b, the difference became insignificant. This result suggests that western German cohabiting women were indeed more negatively selected than their eastern German counterparts. However, adding information on religion and economic activity in Model 3b increased the stability difference again to a significance level of *p* < 0.01. The regional risk differential became even more pronounced in Model 4b, which accounted for the protective effect of further children and parental stability, while considering unobserved heterogeneity in Model 5b did not change the model results.

With regard to marriage, Model 1b replicated the prior finding of Model 1a by showing that there was a significantly higher degree of union stability among women who were married when they had their first child. Eastern German married women were no longer shown to have had a higher degree of union stability than cohabiting women in Model 2b, which might indicate that selection into marriage explains the risk differentials. Conditional on the higher level of secularization in eastern Germany, the risk of separation among eastern German cohabiting women was no longer found to have differed from that of western German married women (Model 3b). Hence, the lower separation risk among western German married women can be explained by their lower level of secularity. The interaction of region and marital status information demonstrated that the determining influence of the union context at the birth of the first child could be attributed to the observed characteristics in the case of eastern German mothers. This was clearly not the case for western German mothers, who showed marked stability differences by marital status at the birth of their first child throughout Model 1b to Model 4b. Only by accounting for unobserved characteristics was it possible to explain the separation risk differentials by marital status.^6^

**Fig. 3:**
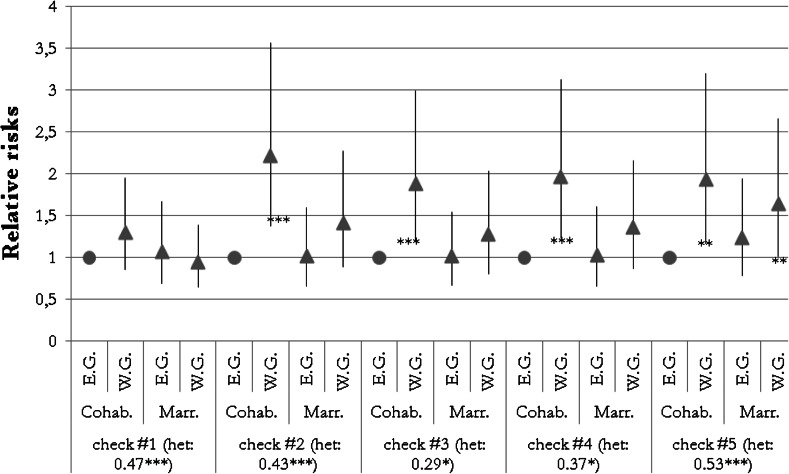
Robustness checks; results of the interaction of the region and the union form at the time the first child was born, shown in relative risks with 95 % confidence intervals and significance levels, the results of residual terms are shown in beta coefficients. *Sources* pairfam/DemoDiff (2008–2011), own estimates. Significance levels ***p < 0.01; **p < 0.05; *p < 0.10. *E.G.* Eastern German women, *W.G.* Western German women, *Cohab.* cohabiting at first childbirth, *Marr.* married at first childbirth. *Notes* All models based on model 5b. Robustness check #1: Model 5b without controlling for religious affiliation; Robustness check #2: Model 5b without controlling for union duration prior to childbirth; Robustness check #3: Model 5b, without coefficients that were insignificant; Robustness check #4: Model 5b, residual variance of hazard model fixed to 0.8; Robustness check #5: Model 5b, residual variance of hazard model fixed to 1.2

Several checks were conducted to test the robustness of the results. First, I excluded in separate estimations the control for religious affiliation (check #1) and union duration prior to childbirth (check #2). Second, I removed independent variables from the equations that did not significantly influence the outcome, referring to the results in Model 4a (check #3). Third, the variance of *ε* was fixed to 0.8 (check #4) and 1.2 (check #5). The results are shown in Fig. 3. In no cases did the omission influence the sign of the correlation coefficient. Western German cohabiting mothers were shown to have had significantly elevated risks of separation in all of the models, except in the model that did not account for religion. This demonstrates that religion is an important factor in the union stability of first-time parents.

*Model 1b* controlled for the region and union form at the time the first child was born (interaction), the piecewise continuous baseline (the age of the first child) and birth cohorts. *Model 2b* controlled for the region and the union form at the time the first child was born (interaction), the piecewise continuous baseline (the age of the first child), birth cohorts, school education, the union duration prior to the first childbirth, and the age when the first child was born (linear and squared). *Model 3b* controlled for the region and the union form at the time the first child was born (interaction), the piecewise continuous baseline (the age of the first child), birth cohorts, school education, the union duration prior to the first childbirth, the age when the first child was born (linear and squared), religious affiliation, and economic activity. *Model 4b* controlled for the region and union form at the time the first child was born (interaction), the piecewise continuous baseline (the age of the first child), birth cohorts, school education, union duration prior to the first childbirth, the age when the first child was born (linear and squared), religious affiliation, economic activity, parental separation, the number of biological children, the partnership order, whether the child was born before the custody reform in 1998, the first child’s characteristics (sex, health, season of birth). Model *5b* controlled for the region and the union form at the time the first child was born (interaction), the piecewise continuous baseline (the age of the first child), birth cohorts, school education, union duration prior to the first childbirth, the age when the first child was born (linear and squared), religious affiliation, economic activity, parental separation, the number of biological children, the partnership order, whether the child was born before the custody reform in 1998, the first child’s characteristics (sex, health, season of birth), and the unobserved selection into childbearing within cohabitation.

## Conclusion

In the past, most of the research on the determinants of union stability has concentrated on the dissolution rates of cohabitations in comparison to marriages. Relatively little is known about how the union stability of cohabitations differs in different contexts, and little attention has been paid to the relationship between the prevalence of births among cohabiting couples and the separation behavior of the parents. If childbearing within cohabitation increases and the separation rates of cohabitations remain stable, this would lead to higher overall separation levels of unions with children involved, and consequently, to a higher prevalence of lone-parenthood and step-families.

This study investigated the impact of cohabitation on the union stability of young parents in a comparative perspective; namely, in eastern and in western Germany. Childbearing within cohabitation has traditionally been higher in eastern than in western Germany. The study showed that even among mothers who started their reproductive careers in reunified Germany, childbearing within cohabitation was much more common among eastern than among western Germans: among mothers of the birth cohorts 1971–1973 and 1981–1983, 60 % of eastern German partnered women had their first child while cohabiting, compared with only 27 % of western Germans.

The study opened with two opposing hypotheses with regard to the relative union stability of cohabiting mothers in eastern and western Germany. On the one hand, the higher share of childbearing within cohabitation in eastern Germany may have reduced the share of negatively selected couples among cohabiting parents, which should in turn have improved union stability. On the other hand, both eastern and western German cohabiting mothers were assumed to have relatively liberal attitudes, which may be reflected in, for example, their high levels of secularization and full-time employment, and, consequently, in their levels union stability.

The study showed that eastern German cohabiting women had better prospects of partnership success than western German cohabiting women. Indeed, the former group had, on average, more education and a longer union duration prior to giving birth than the latter group, and these characteristics are generally assumed to reduce negative selectivity. However, among mothers of the birth cohorts 1971–1973, eastern Germans were younger at the time their first child was born, which should be negatively related to match quality, and consequently, to union stability. The results demonstrated that the shorter union duration in particular helps to explain the higher risk of separation among western German cohabiting mothers. This suggests that western German couples may have had insufficient time to screen their partners. Less compatible partners were “weeded out” to a lesser extent before family formation took place, which increased the risk of separation afterward.

Eastern and western German mothers differed in other respects as well. Eastern German cohabiting mothers were found to have more non-traditional values, because they were less religious and were more likely to have been in full-time employment after having a child than western Germans. This also applied to their married counterparts. A comparison of the probit and the hazard model results revealed that, in addition to a short union duration, the lack of a religious background was the main selective factor that promoted childbearing within cohabitation and increased the risk of separation. When religious affiliation was controlled for, the risk of separation among western German cohabiting mothers was shown to have been almost twice as high as among eastern Germans. This decomposition effect seems to be related to the separation risks associated with the religious background, the marriage timing, and the prevalence of secularization: the results showed that, in general, the lack of church affiliation increased the risk of separation. The Christian church promotes the marital family and life-long marriage, and views non-marital living arrangements as inferior or even unacceptable. This explains why traditional values are related to a lower risk of separation among marital unions, while they do not protect non-marital unions (Schnor 2012). However, the union context at the time the first child was born represents only a snapshot in the partnership biography of the parents. If the couple decided to marry after the child’s birth, the religious norms might have become relevant with regard to marital stability. It has been shown that western German women have higher marriage rates than eastern Germans, even after childbirth (Bastin et al. 2012).^7^ As a consequence, accounting for the women’s religious affiliation leads to substantial changes in the estimation coefficients. This suggests that cohabitation is a fragile arrangement that ends either in marriage or in separation among western German parents, while it is a more stable arrangement in eastern Germany (see the categorization of cohabitation by Heuveline and Timberlake (2004) and Perelli-Harris et al. (2012)).

Comparing cohabitation to marriage, the study found that selection appears to be the main explanation for the higher separation rates among women who cohabited at the time of their first childbirth. However, selection mechanisms worked differently among eastern and western German mothers. In the eastern German case, the shorter union duration of cohabiting mothers was the main explanation for why they had a higher degree of union instability than married mothers. In western Germany, women who cohabited were in less stable unions than women who were married, as long as unobserved factors were not considered. Unmeasured partnership characteristics may have influenced these processes. These findings suggest that cohabitation and marriage differ in many more respects in western Germany than in eastern Germany. The results further indicate that childbearing within cohabitation may be perceived differently, and that the perception of cohabitation as an equivalent to marriage is restricted to eastern Germany.

Overall, no significant differences were found in the levels of union stability of eastern German and western German first-time parents (see again Model 0–2a), despite the large difference in cohabitation levels. This signals that policymakers should not be concerned about the increase in non-traditional family forms, as this development does not necessarily mean a decline in the nuclear family. Thus, policies that seek to prevent families from breaking up by promoting marriage are likely to be inefficient. The duration of a couple’s union before they start a family has a much greater influence on the stability of their partnership than their marital status.

“The selection argument is used mainly to explain the lower marital stability of people who cohabited prior to marriage but it can also be applied to explain the high union instability of current cohabitors,” Liefbroer and Dourleijn have observed (2006). The present study followed this statement, and tried to adapt the methodological approach to the context of childbearing within cohabitation. Assuming a standard normal distribution in the variances of the probit and hazard residuals, the increased risk of union disruption among women who cohabited at the time their first child was born could be entirely attributed to the selection of the most separation-prone into cohabitation. As the models were based on single-spell data, the variances had to be fixed. However, the identification would be improved if multiple spells were used. Standard multi-process estimations, as presented by Lillard et al. (1995), refer to multi-spell data, and include, for example, higher order marriages to identify the correlation structure. This strategy cannot be easily transferred to the context of non-marital family formation, as this event only occurs once in the individual biography. Including the marital status at the time of higher order births or the union stability of step-families do not seem to be appropriate solutions to this problem, because these events differ substantially from that of first-time parenthood. To test the robustness of the model results in the present study, different fixed values were assigned to the variance.

A drawback of the study is that the data did not include information on the characteristics of her partner or her family of origin. Also of importance for the present investigation is the differing selection of eastern and western Germans into the sample used. The eastern German women were more likely than the western German women to have become mothers and to have had their first child at a young age. The analysis did not completely capture the differences in these characteristics.

What can we learn from this cross-regional comparison? A large number of studies have helped to solidify the view that cohabitation is a very fragile form of partnership. The present investigation has provided new insights into the issue of union stability of cohabiting parents. The study has systematically compared the characteristics of cohabiting mothers living in a context with low shares of childbearing within cohabitation to those of cohabiting mothers living in a context in which the majority of parents have their first child outside of marriage. The German example has shown that the context plays a central role for the union stability of parents. The results have demonstrated that in a context in which cohabitation represents the most common type of union for family formation, the union stability of cohabiting mothers can be high. The study has further shown that unobserved heterogeneity mechanisms, which are usually found to explain the destabilizing influence of premarital cohabitation on marital stability, can be applied to the stability of the unions of parents who started their family while cohabiting. However, the unobserved characteristics of the women who decided not to marry could not explain why cohabiting women had better union prospects in a setting in which their union type was more prevalent. The findings for Germany suggest that the prevalence of childbearing within cohabitation does not drive family instability in general. Further comparative studies are needed to clarify the influence of childbearing patterns on union stability. Recent studies (Liefbroer and Dourleijn 2006; Reinhold 2010; Svarer 2004) have suggested that premarital cohabitation ceases to increase divorce rates when about one-half of the population cohabit. Future comparative research may determine whether this critical mass level is also relevant for the stability of couples who were cohabiting when they had their first child.

## Footnotes

1 The German Family Panel is coordinated by Josef Brüderl, Johannes Huinink, Bernhard Nauck, and Sabine Walper. It is funded as a long-term project by the German Research Foundation (DFG).

2 As West Berlin was affected by West German policies, the proportion of non-marital births was much smaller than in East Berlin (Klüsener and Kreyenfeld 2009). To account for these historical differences, western Berlin is counted as western Germany, although it is situated in the eastern German region.

3 It is a coincidence that these shares are identical to the overall shares of non-marital births given by the German Federal Statistical Office. The latter considers the share of non-marital births relative to *all* live births and includes cohabiting as well as single mothers in 2010. According to these official statistics, the share of non-marital *first* births in 2010 was 37 % among western Germans and 74 % among eastern Germans.

4 Among the women who married in response to the arrival of their first child, 29 % of western Germans and 24 % of eastern Germans married between the third month of pregnancy and the child’s birth.

5 We assumed a standard normal distribution of *ε*. Allowing for changes in the pre-fixed level of the variance of *ε* (from 0.6 to 1.8) did not change this result. Within this range, selection effects explain the higher risk of separation of women who cohabited at first childbirth. Complete results are available upon request from the author.

6 As a significance test, the reference category was changed to western German married women. In Model 4b, western German cohabiting women had a risk of separation that was 74 % higher (*p* < 0.01). In Model 5b, no significant differences in the risk of separation compared to married western German women were found.

7 In the sample of this study, this difference was most pronounced in the first year after childbirth: among those mothers who were not married but who were cohabiting at the time of the first childbirth, 22 % of western Germans and 12 % of eastern Germans married within a year of the first childbirth. Another 32 % in western Germany and 30 % in eastern Germany married at a later point in time.
